# Three dimensional approach to investigating biological effects along energetic ion beam pathways

**DOI:** 10.1038/srep44732

**Published:** 2017-03-15

**Authors:** Xinglin Li, Shuguang Sun, Shanying Wang, Wenjian Li, Ying Qu, Weidong Cui, Tianren Sun, Jian Zhang, Jufang Wang, Guangming Zhou, Shuli Man, Yi Chen, Fuping Lu, Zengquan Wei, Genming Jin

**Affiliations:** 1 Key Lab of Industrial Fermentation Microbiology (Tianjin University of Science and Technology), Ministry of Education, Tianjin, 300457, China; 2 College of Bioengineering, Tianjin University of Science and Technology, Tianjin, 300457, China; 3 Forestry College of Nanjing Forestry University, Nanjin, 210037, China; 4 Institute of Modern Physics, Chinese Academy of Sciences, Lanzhou, 730000, China; 5 Hefei Jiushi Garden Construction Company, Hefei, 230000, China; 6 Forestry College, Anhui Agricultural University, Hefei, 230036, China; 7 Department of Radiological Medicine and Protection, School of Medicine, Suzhou University, Suzhou, 215123, China; 8 Tianjin Academy of Education, Enrollment and Examination, Tianjin, 300060, China

## Abstract

Heavy ion beams have many exciting applications, including radiotherapy of deep-seated tumors and simulation tests of space irradiation for astronauts. These beams often use a feature that concentrates the energy deposition largely along the end of the energy pathway, leading to different distributions of biological effects along the axial direction. Currently, there is relatively little information regarding the radial directional difference of biological effects along the heavy ion paths. This study utilized a filter membrane that was quantatively applied with cells to demonstrate a 3D distribution model of irradiation on biological effects in living organisms. Some results have indicated that there is excitatory effect on the non-irradiated regions with energetic ions, which may give new insights into the distribution of biological effects along the paths of heavy ion beams with mid-high energy.

In comparison to X-rays and γ-rays, heavy ion beam irradiation at both low and high energy has three significant properties. First, energy deposition from heavy ions mainly occurs near the end of the path when traveling through matter^1^,^2^,^3^. Second, a heavy ion has high linear energy transfer (LET)^4^,^5^,^6^, especially at the peak of the Bragg curve. For example, irradiation with 90 MeV/uncleon ^12^C^6+^ results in LET = 40 keV/μm in the plateau region, while a LET = 189 ± 15 keV/μm is observed in the Bragg curve peak. Third, heavy ion irradiation of biomaterials has high relative biological effectiveness due to optimal LET features^4^,^5^,^6^,^7^,^8^. Thus, heavy ion beams at both low and mid-high energy have broad and exciting applications.

Low energy heavy ions are widely used to introduce genetic mutations into animals^9^, plants^10^,^11^,^12^, and microorganisms^13^,^14^,^15^, as well as in performing molecule modificatons^16^,^17^ and exogenous gene transformation^18^,^19^,^20^. Mid-high energy heavy ions could be used in novel radiotherapies aimed to disrupt deep-seated tumors, based on the breakout-peak of the Bragg curve^21^,^22^,^23^,^24^,^25^,^26^, while these ions could also be used to explore safe measures against space irradiation of astronauts during space travel^27^,^28^,^29^. In terms of inducing mechanisms, in addition to energy (mass) deposition^1^,^2^,^3^, mid-high heavy ion beams that travel through matter are also characterized by charge exchange, momentum transfer, and secondary irradiation^30^,^31^,^32^,^33^,^34^,^35^. Although different biological effects are observed in both the axial and radial directions along the path of heavy ion beams, the biological effects of heavy ion beams along the radial direction have yet to be well elucidated. This is likely due to two reasons: 1) biological effects in the axial direction are considered to be more important than in the radial direction and 2) There is no simple and effective approach in assaying biological effects along the radial direction. Currently, little is known regarding the biological effects along the radial direction of heavy ion beam paths, excluding the distribution of the energy (mass) deposition^36^,^37^.

In this study, a filter membrane was treated with living cells, rolled into a cylinder, irradiated with heavy ion beams, and cut into individual assay units after unwinding the cylinder. The biological effects along both the axial and radial directions was used to combine the assay units to establish a 3D distribution model of biological effects along the pathway of mid-high energy heavy ion beams.

## Results

### Overview

**Figure 1:**
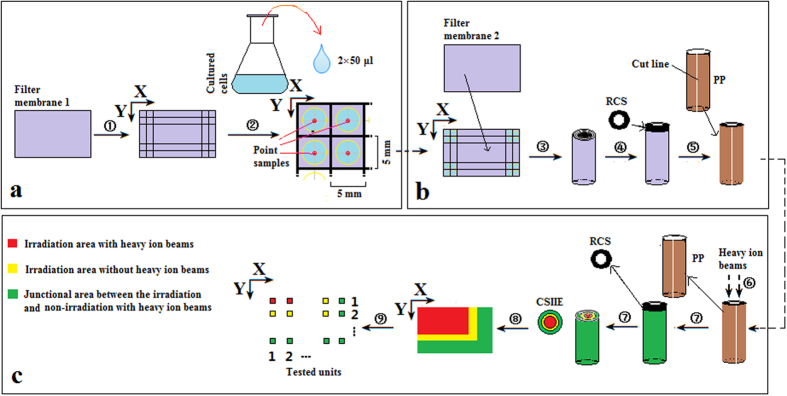
Visualization of 3D experiment with heavy ion beams. (**a**) Sample preparation: The piece of filter membrane was delineated in 5 × 5 mm sections with a pencil (Step 1), which was then sterilized; Cells at logarithmic phase of growth were quantitatively applied to the middle of each section on the membrane (Step 2) and dried with sterile absorbent paper (not shown in Fig. 1). (**b**) Irradiation target preparation: a second piece of filter membrane was placed onto the sample membrane, and the two were tightly wrapped into a cylinder together (Step 3), subsequently, we used a parafilm to seal the cylinder (not shown in Fig. 1). The cylinder was affixed to a copper sheet with a round hole (Step 4) and a plastic pipe with a incision was used to fix the cylinder (step 5) for storage at 4 °C. (**c**) Irradiation and preparation of the tested units: The membranes and copper sheet were irradiated with mid-high energy heavy ion beams (Step 6). Under sterile conditions, the plastic pipe and copper sheet were removed (Step 7) and the filter membrane opened (Step 8). Based on the experimental design, the filter membrane was cut into assay units along the corresponding sections lines, and numbered by assay unit (Step 9). All experiments were performed according to the assay units. CSIIE, Cross section of irradiation at initiative end; PP, Plastic pipe; RCS, Round copper sheet with a round hole at the centre; X, radial direction; Y, axial direction (i.e. irradiation direction with heavy ion beams).

The 3D sample treatment and analysis consisted of four steps: sample preparation, irradiation target preparation, irradiation with heavy ion beams and assay unit preparation (Fig. 1).

Cells were cultured to logarithmic growth phase in liquid culture and prepared for analysis in subsequent steps. A filter membrane (FM1, Φ0.22-μm pores) was sectioned into 5 × 5 mm-size grids with a pencil, then both the FM and another filter membrane (FM2)of the same size were encapsulated in kraft paper to sterilize (not shown in Fig. 1), respectively. Under sterile conditions, each grid of the FM1 was treated twice with 50 μL of a cell suspension with a multiple-micro injector and the FM2 was used to cover the FM1 sample into a FM-cell layer-FM (FM-CL-FM). And the FM-CL-FM was dried with sterile absorption paper(not shown in Fig. 1) to absorbe the redundant water from the sample (Fig. 1a).

The absorption paper was removed after 15 min (not shown in Fig. 1), the FM-CL-FM was rolled into a cylinder, and the cylinder was sealed with a parafilm (not shown in Fig. 1) and a hard plastic pipe (PP) for storage at 0–4 °C in turn. The cell layers of the FM-CL-FM treated with *Chlorella vulgaris (C. vulgaris*) or *Saccharomyces cerevisiae (S. cerevisiae*) cells had an average thickness of 0.20 mm (Range: 0.15–0.25 mm). In order to explore the biological effects in the non-irradiated regions (i.e. bystander effects or indirect effects), a round copper sheet with a circular hole in the center site was added to one end of the cylinder before it was sealed with the PP (Fig. 1b).

The end of the cylinder without the copper sheet was opened and treated with liquid medium between the cylinder layer gaps prior to irradiation, and then the cylinder was resealed and placed under the irradiation window, in which the copper sheet end of the cylinder was vertically placed along the heavy ion beam projection. The cylinder was irradiated with 80 MeV/u ^12^C^6+^ at a dose rate of 0.5 Gy/min at the Heavy Ion Research Facility in Lanzhou (HIRFL, Institute of Modern Physics, Chinese Academy of Science, Lanzhou, China), using a 40-mm irradiation diameter. The carbon ion beam production and dosimetry has been described previously^38^. In this study, two kinds of irradiation doses were used: 20 Gy and 500 Gy (Fig. 1c).

**Figure 2:**
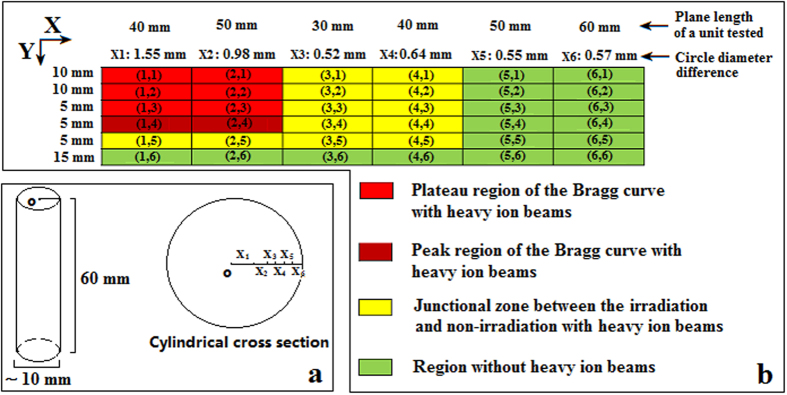
Assay unit segmentation and corresponding coordinates treated with 80 MeV/u ^12^C^6+^ irradiation in a 10-mm diameter window. (**a**) Cylindrical cross section; (**b**) Assay unit segmentation and corresponding coordinates. **X**_**1**_, radius of the cylinder core area; **X**_**2-6**_, cycle thickness mean of an assay unit in the cylinder.

After irradiation, the irradiated cylinder was immediately opened to remove the PP, the RCS and the parafilm, following by unwinding the cylinder to access the irradiated FM-CL-FM. The irradiated FM-CL-FM was cut into assay units along the grided lines, of which each assay unit consisted of a 5 × 5-mm section (see Fig. 1c) or some sections (such as this study, see Fig. 2b). Each assay unit contained the quantitative cells (100 μL of the cultured liquid per grid) and was used to directly assess biological effects and/or to examine the effects of irradiation after subculturing (Fig. 1c).

Generally, x and y coordinates can be used to represent the position of any assay unit respective to the beginning and end along the axial and radial directions of heavy ion beam irradiation, revealing 3D biological effects after combining these indexes from all assay units. For example, the coordinate (1, 1) represents the first assay unit along the initiating end from the plataeu region of the Bragg curve, which was located at the beginning of the axial irradiation.

In this study, irradiation was performed using 80 MeV/u ^12^C^6+^ at the 0.5 Gy/min dose. Based on theoretical calculations (Lise^++^9.9: http://lise.nscl.msu.edu/lise.html) and the measurement of CR-39 heavy ion detectors^39^,^40^, the Bragg peak of energy loss from water appeared at 25–30 mm along the axial orientation of ion beam track. As a result, a new cylinder (60 mm high and ~10 mm in diameter) was utilized with 270 × 60 mm dimensions (Fig. 2). The cylinder was cut into 6 × 6 assay units after irradiation and correlated to their corresponding coordinates. The cylindrical diameter and height, as well as thickness per assay unit are shown in Fig. 2a and b, respectively.

### 3D distribution display of survival rate

**Figure 3:**
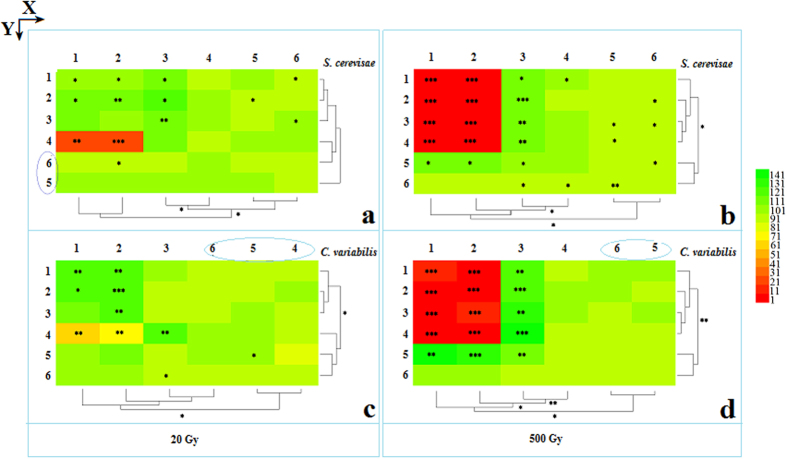
Analysis of survival rate with 80 MeV/u ^12^C^6+^ irradiation. (**a**) *S. cerevisae* cells irradiated at a 20 Gy dose. (**b**) *S. cerevisae* cells irradiated with a 500 Gy dose. (**c**) *C. vulgaris* cells irradiated with a 20 Gy dose. (**d**) *C. vulgaris* cells irradiated with a 500 Gy dose. Heatmap and cluster analyses were performed according to the heatmap method (*HemI 1.0.1—Heatmap Illustrator*) described by Deng W. *et al*.^43^. In this paper, option parameters were as follows: Normalization, Linear (default); Clustering, Hierarchical—Average linkage (default); Similarity Metric, Pearson distance (default); Bar, Discrete bar. Statistical significance between each experimental group and the control is denoted as: *Represents p < 0.05, **p < 0.01, and ***p < 0.001.

Low dose irradiation (20 Gy) was compared to control treatments (i.e. non-irradiated treatments, 100% survival rate), which revealed that the survival rate of irradiated groups had differential expression patterns. There were three significant changes (p < 0.05, Fig. 3a and c). First, a direct excitatory effect (DEE) was produced from the heavy ion beams at low doses, which were noted in the plateau regions of the Bragg curves: (1, 1)-(2, 3), consisting of 6 assay units. Second, an indirect excitatory effect (iDEE) was produced from the heavy ion beams, which occurred in the boundary regions proximal to the ion beam paths: (3, 1)-(3, 3) and (1, 5)-(3, 5), consisting of 7 assay units. Three, significant damage effect (SDE), was noted in the Bragg curve peak: (1, 4) and (2, 4), consisting of 2 assay units. Comparison of the two kinds of cells revealed that iDEE and SDE from *S. cerevisae* (Fig. 3a) were more significant than the data from *C. variabilis* (Fig. 3c), while the DEE from *C. variabilis* cells was more significant than that of *S. cerevisae* cells.

High dose irradiation (500 Gy) was compared to control treatments, and 8 assay units from both the plateau region and the peak of Bragg curves demonstrated SDEs (Fig. 3b and d for *S. cerevisae* and *C. vulgaris*, respectively), while the boundary regions of the heavy ion beams paths: (3, 1)-(3, 3) and (1, 5)-(3, 5), revealed iDEEs for high doses, rather than iDEE as described previously.

In comparing the two doses, it was revealed that the iDEEs of cells irradiated with high dose heavy ion beams (radial depth into non-irradiation regions; about 0.52 + 0.64 = 1.16 mm, Fig. 2b) had more significant changes than cells irradiated with low dose ion beams (radial depth into non-irradiation regions: about 0.52 mm, Fig. 2b).

### Distribution of superoxide anion radicals

**Figure 4:**
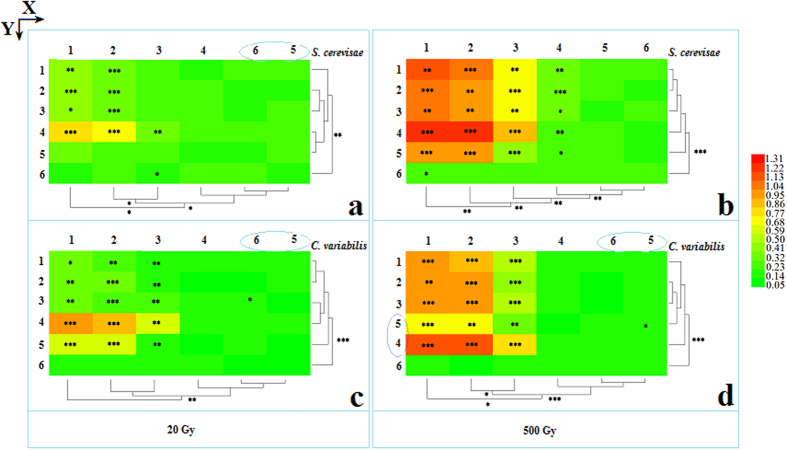
Analysis of superoxide anion radicals (OD_530_) after irradiation with 80 MeV/u ^12^C^6+^. (**a**) *S. cerevisae* cells irradiated with a 20 Gy dose. (**b**) *S. cerevisae* cells irradiated with a 500 Gy dose. (**c**) *C. vulgaris* cells irradiated with a 20 Gy dose. (**d**) *C. vulgaris* cells irradiated with a 500 Gy dose. Heatmap and cluster analyses were performed according to the heatmap method (*HemI 1.0.1—Heatmap Illustrator*) described by Deng W. *et al*.^43^. In this paper, option parameters were as follows. Normalization, Linear (default); Clustering, Hierarchical—Average linkage (default); Similarity Metric, Pearson distance (default); Bar, Discrete bar. Statistical significance between each experimental group and the control is denoted as: *Representsp < 0.05, **p < 0.01, ***p < 0.001.

Superoxide anion radical concentrations were measured for low dose irradiation treatments (20 Gy) for both *S. cerevisae* and *C. vulgaris* (Fig. 4a and c, respectively). Compared with the control treatment (OD_530_ of 0.00), the superoxide anion radical concentration was increased in the irradiated groups. Similarly, significant changes in superoxide anion radicals were correlated with survival rates based on changing trends and the distribution of the assay units for both kinds of the tested cells. Interestingly, the iDEEs of *C. variabilis* (Fig. 4c) were more significantly changed when compared with those of *S. cerevisae* (Fig. 4a).

High dose irradiation (500 Gy) was also reported for superoxide anion radical concentration for both *S. cerevisae* cells and *C. variabilis* (Fig. 4d). In comparison to control treatments (non-irradiated treatments), significant changes in superoxide anion radicals were also correlated with the survival rates described previously in the changing trends and the assay unit distribution for both kinds of the tested cells. Importantly, the iDEE region range for *S. cerevisae* cells (Fig. 4b) was significantly greater than that of *C. variabilis.* cells (Fig. 4d).

Interestingly, clustering analysis along the y-axis for *S. cerevisae* (Fig. 4d) revealed that there were no significant differences in the superoxide anion radicals between the Bragg curve peak region (*y* = 4) and the plateau region (*y* = 1–3).

In comparing the differences between low and high doses, the radial depth into the non-irradiated sample was observed to be ~0.52 mm (Fig. 2b) with a high dose of heavy ion beam irradiation related to superoxide anion radical formation, while the radial depth into the non-irradiation region was ~0 mm using low dose heavy ion beam irradiation.

## Discussion

In this study, two endpoints were utilized to reveal the biological effects derived from irradiation of heavy ion beams: survival rate and superoxide anion radical formation. The survival rate of the irradiation cells may exhibit some features for humans in either theory or on application. In addition, the presence of superoxide anion radicals is the prominent radical in biological cells that is caused by irradiation treatment, and is also the precursor molecule for oxygen radicals^41^, which can then be transformed directly into other oxygen radicals. Thus, the biological effects of irradiation with heavy ion beams needs to be tested, especially by capturing and testing two endpoints from living samples.

**Figure 5:**
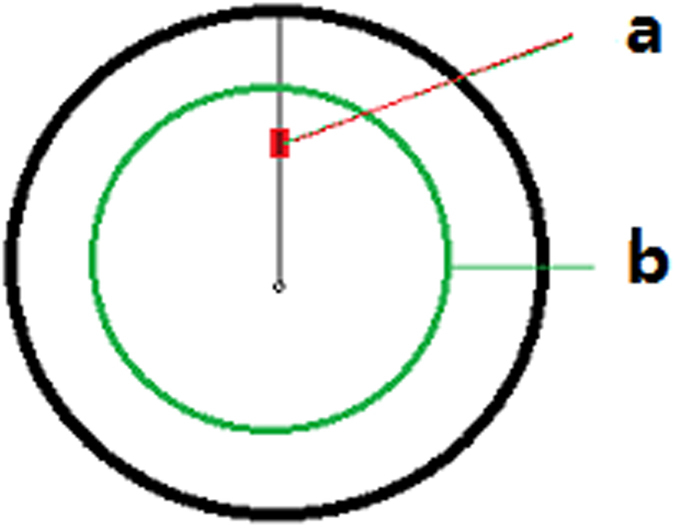
Comparison of two sampling methods. (**a**) Sampling method using the diameter difference. (**b**) Sampling method using the perimeter.

In order to investigate the energy (mass) deposition of heavy ions along the radial direction, Shuichi, T. *et al*. used a simple method whereby an irradiated sheet was cut into cross-sections along the radial direction^36^,^37^,^38^ (Fig. 5). Herein, assay units were sampled along circular lines at a 0.2 mm width using a plane-cylinder-plane method with FM-CL-FM, reducing the sampling error when compared to previous methods (i.e. the diameter difference sampling) along the radial direction (Fig. 5 and Table 1). This method meets sampling requirements for simple operation, small error and high efficiency, which is convenient for investigating the 3D biological effects of heavy ion beam irradiation. The cylinder was partially covered with a copper sheet prior to irradiation with heavy ion beams, and three simultaneous biological effect studies were performed for directional effects (irradiated regions with heavy ions), indirection effects (testing cells from the non-irradiated regions with heavy ions) and bystander effects (testing non-irradiated cells with heavy ions only after irradiation or treating non-irradiated cells with cells and/or medium from irradiated regions).

In order to measure the indirectional effects of heavy ion beams, FM-CL-FM was rolled into a cylinder with an about 10-mm diameter and covered with a 5-mm thick copper sheet at the initiating end of the cylinder, based on the energy calculation of 100 MeV/u ^12^C^6+^ (Table 2), which directly faced the irradiation window. Using ion beams at 100 MeV/u, energy levels of charged particles resulted from the secondary irradiation, which was <10 MeV/u. The penetration depth of charged secondary particles was <1 mm in water (Table 2), thus limiting the irradiation depth of secondary particles in the radial direction. When a 0.5 mm of radial thickness was used to test the radial effects, which is equivalent to two rotations about the cylinder with an about 10-mm diameter, a sufficient number of samples treated with secondary particles along the radial direction were used compared to the method reported by Shuichi Tsuda, *et al*.^36^.

**Figure 6:**
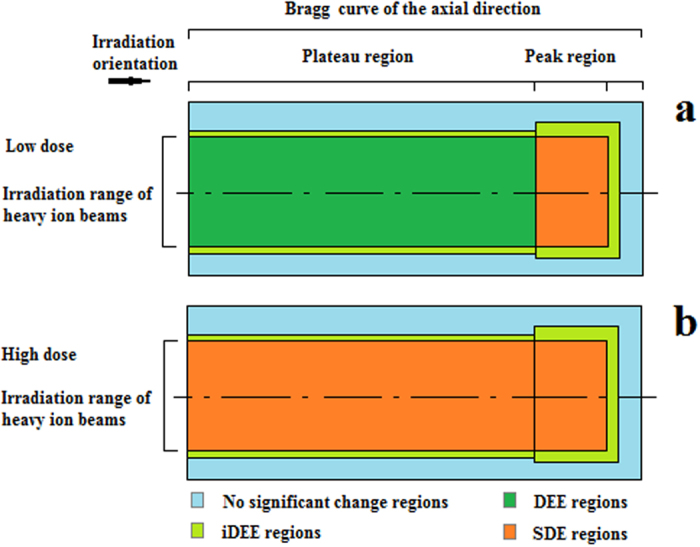
Sectional view revealing 3D biological effects along the axis of the irradiated cylinder. (**a**) Low dose irradiation. (**b**) High dose irradiation. iDEE, indirect excitatory effect; DEE, direct excitatory effect; SDE, significant damage effect.

In summary, two different 3D distribution models could be used to explain the differences in irradiation doses with heavy ion beams. For low dose irradiation, the excitatory effect occurred both in the plateau region (DEE) and in the boundary region close to the Bragg curve within the axial direction (iDEE; Fig. 6a). For high dose irradiation, the excitatory effect occurred only in the boundary region of the heavy ion pathway (iDEE; Fig. 6b). In addition, there was SDE on the survival rate at low doses of energetic heavy ions, suggesting a new understanding in the “saddle-like” dose-response curve that represents the survival rate. These results may provide new insights into the different distributions of 3D biological effects along the pathways of heavy ion beams at mid-high energy.

## Methods

### Cells and cell medium

*C. vulgaris* and *S. cerevisiae* were obtained from the Plant Cell Engineering Lab., Bioengineering College, Tianjin University of Science and technology. *S. cerevisiae* cells were cultured in a medium composed of 20 g/L peptone, 10 g/L yeast extract, and 2 g/L glucose (pH 7.0). *C. vulgaris* cells were cultured in a medium composed of 0.02 g/L Na_2_CO_3_, 2.0 g/L NaNO_3_, 0.02 g/L KH_2_PO_4_, 0.1 g/L MgSO_4_, and 0.8 g/L urea (pH 6.0).

### Shaking culture

The cells were inoculated into liquid medium and shaken at 120 rpm at 25 °C for ten days (*C. variabilis*) or at 28 °C for 20 hours (*S. cerevisae*), which corresponded to logarithmic speed growth phase. The cell density was then adjusted to 1 × 10^9^ cells/mL with fresh medium, such that each 100 μL of culture contained 1 × 10^8^ cells/per grid on the FM.

### Plate culture

For each assay unit, cells were spread-plated on solid medium in 110-mm Petri dishes, achieving a total of 100–200 cells on each plate. Subsequently, the Petri dishes were placed into incubators to culture the cells at 25 °C for 10 days (*C. variabilis*) or at 28 °C for 20 hours (*S. cerevisae*).

### Irradiation of cells with carbon ion beams

Cylinders were exposed irradiation with 80 MeV/u ^12^C^6+^ at a dose rate of 0.5 Gy/min, which was performed at the Heavy Ion Research Facility in Lanzhou (HIRFL, Institute of Modern Physics, Chinese Academy of Science, Lanzhou, China). The irradiation diameter was 40 mm. The carbon ion beam production and dosimetry has been described previously^38^. Two irradiation doses were used: 20 and 500 Gy.

### Survival rate determination

After irradiation, cells from each assay unit were cultured in liquid medium as described previously. Then, 100 μL of culture, containing ~1 × 10^3^ cells from each assay unit, was spread onto solid and incubated at 25 °C for ten days (*C. variabilis*) or at 28 °C for three days (*S. cerevisae*), which was repeated three times. Survival rates were calculated in comparison to non-irradiated cells as follows.


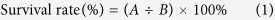


*A* represents colony numbers from the irradiation group, while *B* represents colony numbers from the non-irradiated/control group.

### Cell extract preparation

10-mL centrifugation tubes were sterilized, dried and weighed, then used to centrifuge 4-mL of logarithmically-growing cells at 7,000 rpm at 4 °C for 15 min. The supernatant was removed, the cell pellet + tube weighed and the cell mass determined. If the original cell culture density was relatively dilute, the previous steps were repeated to attain enough cells for analysis. Subsequently, cells were suspended in 100 mM phosphate buffer (pH 7.8) to a 0.2 mg/μL concentration. In an ice bath, the tested cells were ground into a homogenous mixture with blunt-pointed tweezers. The homogenized cell suspension was centrifuged at 10,000 rpm at 4 °C for 20 min, and supernatant was extracted as the enzyme extract, which was stored at 0–4 °C for subsequent analysis^42^.

### Determination of superoxide anion radical

A modified hydroxylamine oxidation method was used to determine superoxide anion radical concentration. A 8 × 12-hole enzyme-marking-plate that was air-dried was used to perform the experiments, where each hole represented one sample. All reagents and the protocol for hydroxylamine oxidation determination were performed as described by Xiao, H. S. *et al*.^42^. The OD_530_ of the irradiated and non-irradiated groups was utilized to directly measure the superoxide anion radical concentration.

### Data analysis

All data were calculated as mean ± standard deviation. Statistical significances between the experimental and control groups were performed by Student’s t test, where only p < 0.05 was considered to be a statistically significant difference. Cluster analysis was performed according to the heatmap method (*HemI 1.0.1—Heatmap Illustrator*), as described by Deng W. *et al*.^43^. In this paper, option parameters were as follows:

Normalization: Linear (default)

Clustering: Hierarchical—Average linkage (default)

Similarity Metric: Pearson distance (default)

Bar: Discrete bar.

## Additional Information

**How to cite this article**: Li, X. *et al*. Three dimensional approach to investigating biological effects along energetic ion beam pathways. *Sci. Rep.* **7**, 44732; doi: 10.1038/srep44732 (2017).

**Publisher's note:** Springer Nature remains neutral with regard to jurisdictional claims in published maps and institutional affiliations.

## Figures and Tables

**Table 1:** Comparison of two sampling approaches (for ø 35 mm).

Distance from the axis/mm	Perimeter/mm	Sample thickness (diameter difference)/mm	Ratio of perimeter to diameter difference
3	18.8	0.20	94
5	31.4	0.20	157
7	44.0	0.20	220
9	56.5	0.20	283
12	75.4	0.20	377
15	94.2	0.20	471
17.5	109.9	0.20	550

**Table 2:** The implantation depth of the energetic particle into three types of matter (Lise^++^9.9: http://lise.nscl.msu.edu/lise.html).

Particle species	Single nuclear power/MeV/u	Implantation depth in water/mm	Implantation depth in lead/mm	Implantation depth in copper/mm
^12^C^6+^	100	25.6	3.8	4.5
^12^C^6+^	200	85.8	12.2	14.6
B (Secondary particle)	10	0.51	0.10	0.11
Be (Secondary particle)	10	0.63	0.12	0.13
Li (Secondary particle)	10	0.81	0.15	0.17
He (Secondary particle)	10	1.2	0.41	0.32
H (Secondary particle)	10	1.3	0.42	0.33
